# Initial non-adherence to lipid-lowering medication: a systematic literature review

**DOI:** 10.1186/s12875-024-02524-z

**Published:** 2024-08-05

**Authors:** Catherine E. Cooke, Teisha Robertson

**Affiliations:** 1 Department of Practice, Sciences and Health Outcomes Research, University of Maryland School of Pharmacy, 20 N. Pine Street, Office S446, Baltimore, Maryland, MD 21201 USA; 2 Pharmacy Operations Division, Defense Health Agency, 7700 Arlington Blvd Falls Church, Virginia, 22042 USA

**Keywords:** Hyperlipidemia, Anticholesterolemic agents, First fill failure, Primary medication adherence

## Abstract

**Background:**

The impact on cardiovascular health is lost when a patient does not obtain a newly prescribed lipid-lowering medication, a situation termed “initial medication nonadherence” (IMN). This research summarizes the published evidence on the prevalence, associated factors, consequences, and solutions for IMN to prescribed lipid-lowering medication in the United States.

**Methods:**

A systematic literature search using PubMed and Google Scholar, along with screening citations of systematic reviews, identified articles published from 2010 to 2021. Studies reporting results of IMN to lipid-lowering medications were included. Studies that evaluated non-adult or non-US populations, used weaker study designs (e.g., case series), or were not written in English were excluded.

**Results:**

There were 19 articles/18 studies that met inclusion and exclusion criteria. Estimates of the prevalence of IMN to newly prescribed lipid-lowering medications ranged from 10 to 18.2% of patients and 1.4–43.8% of prescriptions (*n* = 9 studies). Three studies reported prescriber and patient characteristics associated with IMN. Hispanic ethnicity, Black race, lower Charlson Comorbidity Index score and no ED visits or hospitalization were associated with IMN. Lipid lowering prescriptions from primary care providers were also associated with IMN. Four studies described patient-reported reasons for IMN, including preference for lifestyle modifications, lack of perceived need, and side effect concerns. Four intervention studies reported mixed results with automated calls, live calls, or letters. One study reported worse clinical outcomes in patients with IMN: higher levels of low-density lipoprotein and greater risk of emergency department visits.

**Conclusions:**

Up to one-fifth of patients fail to obtain a newly prescribed lipid-lowering medication but there is limited information about the clinical consequences. Future research should assess outcomes and determine cost-effective approaches to address IMN to lipid-lowering therapy.

## Background

Lipid-lowering medications are prescribed to mitigate the risk of cardiovascular disease [1]. The impact of these efforts is lost when the newly prescribed medication is not obtained by the patient. If a patient does not obtain the first fill of the initial prescription, this type of medication nonadherence is referred to as “first-fill failure”, “early nonadherence”, “primary nonadherence” or “initial nonadherence”. Several terms have been used to discuss this type of nonadherence with the International Society for Pharmacoeconomics & Outcomes Research recommending initial medication adherence. Thus, we use the term initial medication nonadherence (IMN) throughout.

Much of the nonadherence research has focused on behavior after a patient begins taking the medication, which is often referred to as “secondary medication nonadherence”. Research on secondary medication nonadherence is abundant and has provided insight into how often patients discontinue refilling their lipid-lowering medication, factors associated with this behavior, and the associated outcomes of nonadherence [2]. The literature has advanced to include systematic reviews of solutions for secondary nonadherence to lipid-lowering medications [3, 4].

Compared to data on secondary medication nonadherence, information about IMN to lipid-lowering medications is scant but growing. The recent growth is due in large part to electronic prescribing supplanting handwritten prescriptions. Electronic prescription data can be linked to other data sources such as prescription claims and pharmacy records to facilitate comparison of what was prescribed to that obtained.

Based on limited research, IMN appears to be common. Internationally, IMN to lipid-lowering medication is estimated to affect about 1 in 5 patients, with higher rates in North America than in Europe [5]. Due to the differences in health care systems and other factors in North American countries, there is a need to further understand IMN to lipid-lowering medication. In addition, prior literature has not focused solely on lipid-lowering therapy. Thus, the objective of this study is to describe the evidence on the prevalence, predictors, outcomes, and solutions for IMN to lipid-lowering medications in the United States.

## Methods

The reporting of this systematic review was guided by the standards of the Preferred Reporting Items for Systematic Review and Meta-Analysis (PRISMA) Statement [6]. A literature search was conducted in Medline (PubMed) from January 1, 2010 to December 31, 2021 to identify articles about IMN using the following search terms: (1) “medication adherence” (MeSH term) combined with “first fill,” “early,” “primary,” or “initial” (in the title or abstract), or (2) “prescription abandonment” [7]. The search was limited to papers published in English and author affiliation within the US. The start date for the search, 2010, was chosen to provide a more recent estimate of real-world experience with IMN to lipid-lowering. Two reviewers screened titles and abstracts of the combined searches and excluded studies in non-US or pediatric populations and those on ongoing nonadherence. Case studies, case reports, and articles that were not studies (e.g., review articles, letters to the editor, commentaries) were also excluded. The full text for articles not excluded by both reviewers was obtained. During this stage, there was an additional requirement for inclusion: the study had to be relevant to IMN to lipid-lowering medications. The reference lists of systematic reviews from the combined searches were also examined to determine whether they included studies that were relevant to IMN to lipid-lowering medications. Additional searches were conducted in Google Scholar using the same search terms as used for Medline search. Discussion between the reviewers resolved discordances in article selection, and the final list of articles included in this review was agreed upon by both reviewers.

## Results

**Fig. 1:**
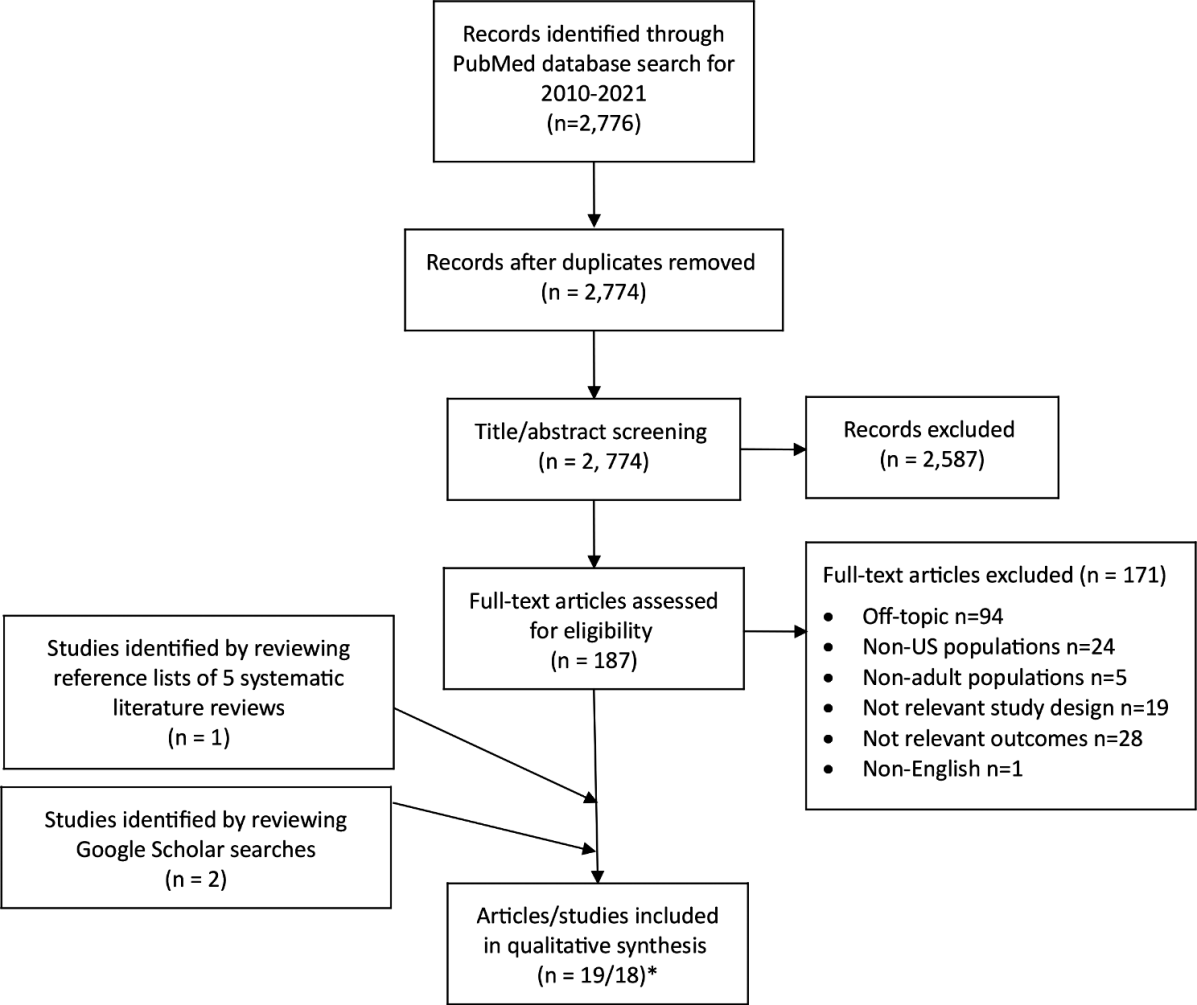
Flowchart of Study Selection. *Two articles described the same study. Figure template adapted from [6]

There were 2,774 unique articles identified from the Medline searches that were screened and reviewed, with 16 articles meeting inclusion and exclusion criteria (Fig. 1**)**. Examining the reference lists of the 5 systematic reviews [8–12] and Google Scholar searches resulted in the inclusion of 3 additional articles, for a total of 19 articles [13–31]. Two of the 19 articles described the same study [18, 19], resulting in 18 studies.

Nine studies analyzed the prevalence of IMN to newly prescribed lipid-lowering medication, [13–22] four studies examined patient-reported reasons for IMN to lipid-lowering medications, [23–26] one study reported outcomes of IMN to statin medications, [27] and four studies assessed interventions to lower IMN [28–31].

Many of the studies assessed IMN to other medication classes [14–22, 24, 29–31] but only the results related to lipid-lowering medications are described.

### Prevalence and predictors of IMN (*n* = 9 studies)

The entire class of lipid-lowering medication was assessed in six studies [14, 15, 17–21] while statins only were examined in three studies [13, 16, 22]. The prevalence of IMN for lipid-lowering medications ranged from 10 to 18.2% of patients and from 1.4 to 43.8% of prescriptions (*n* = 9 studies) (Table 1) [13–22]. These estimates are based on the definition of IMN, which was not obtaining the first fill of either the medication or one in the same class after a new prescription. All but one study defined a new prescription as no prior therapy (same medication or same class) within a certain timeframe (e.g., 6 months). However, when the definition of a new prescription included both new and continuing medications, a lower IMN was found [15]. Out of 69,967 prescriptions for new and continuing medications, the prevalence of IMN was 10.9% compared to 25.2% when only new medications were included.

**Table 1:** Studies reporting prevalence of IMN to newly prescribed lipid-lowering medications (*n* = 9)

Study	Population	Location	Data sources	Medication	New Rx definition	Measurement of IMN	Prevalence of IMN
Cheetham et al., 2013	19,826 patients with a new statin Rx (including combination products) in a group-model managed care organization	Southern California	EMR and pharmacy records	Statin	No statin (including combination products) Rx or refill in the prior 365 days	Did not obtain the medication within 90 days	15.4% (3,049/19,826) of patients
Fischer et al., 2010	3,242 eRxs for new lipid lowering agent in adults from community-based practices	Massachusetts	eRx transactions and pharmacy claims	Lipid lowering agents	No Rx claim for same medication within available prior data (range of 6–12 months prior)	No paid pharmacy claim during study window (range of 1 day to 12 months)	28.2% (913/3,242) of Rxs
Fischer et al., 2011	27,208 eRxs for new antihyperlipidemics from e-prescribing database (In those with any history of Rx claim, 20,429 eRxs for new antihyperlipidemics)	Database available in all 50 US states	eRx transactions and pharmacy claims	Lipid lowering agents	No Rx claim for same medication within prior 6 months	No paid pharmacy claim within 6 months	43.8% ^a^ of Rxs (In those with any history of a Rx, 25.2% ^a^ of Rxs)
Jackson et al., 2014	9,768 new statin eRxs for adults from 100 pharmacies located of a national pharmacy chain	Mid-South region of US	Pharmacy records	Statin	No Rx for the same medication within prior 180 days	Medication or a therapeutic equivalent not obtained not obtained from the pharmacy within 30 days	12.4% (1,209/9,768) of Rxs
Liberman et al. 2010	1,061 new eRxs for dyslipidemia from participating prescribers within a health plan	New Jersey health plan	eRx and pharmacy claims	Lipid lowering agents	No claim for same class within prior 180 days	No claim for eRx or a clinically equivalent medication within 60 days	34.1% (362/1,061) of Rxs
Raebel et al. 2011 & Raebel et al., 2012	4,607 patients with new antihyperlipidemic Rx from an integrated healthcare delivery system	Colorado	EHR and pharmacy records	Lipid lowering agents	No Rx for a medication with the same therapeutic indication in the prior 365 days	Not obtained from the pharmacy (or not transferred to another pharmacy) within 30 days	10% (582/5,848) of patients
Romanelli et al., 2018	3,244 adults with Rxs for lipid-lowering and a completed experience of care survey from a multispecialty ambulatory health care delivery system	California	EHR and pharmacy claims	Lipid lowering agents	New Rx which could be for a new or an ongoing medication	No paid pharmacy claim through expected end date of Rx	18.2% (592/3,244) of patients
Shin et al., 2012	22,249 new lipid-lowering Rxs from an integrated healthcare system	California	EMR and pharmacy records	Lipid lowering agents	No dispensing in the same class within the prior 12 months	Not obtained from the pharmacy within 14 days	22.3% (4,969/22,249) of Rxs
Shrank et al., 2010	405,994 statin Rxs from a national pharmacy chain	National pharmacy chain in US	Pharmacy records	Statin	No Rxs in the same class within the prior 6 months	Did not obtain the Rx or another in the same class at the same or another pharmacy within 30 days	1.4% (5,654/405,994) of Rxs

*Notes*^a^ Absolute numbers not reported

*Abbreviations* EHR, electronic health records; EMR, electronic medical record; e-Rx, electronic prescription; Rx, prescription

Three studies also assessed predictors of IMN to lipid-lowering medications and found age, race/ethnicity, low-density lipoprotein (LDL) level, co-morbidity level, prescription claims history, insurance status, healthcare resource utilization, and prescriber characteristics were associated with IMN [13, 17–19]. In the first study which assessed only statin prescriptions, results from a multivariable logistic regression reported higher likelihood of IMN for older aged prescribers (odds ratio [OR], 1.012, 95% confidence interval [CI], 1.008–1.017) and those considered high volume statin prescribers (OR 1.6, 95% CI 1.4–1.8) [13]. Patients with black race compared to the reference of non-Hispanic White were also associated with a higher odds of IMN (OR 1.3, 95% CI 1.1–1.5). Lower odds of IMN were found with higher patient age (OR 0.991, 0.988–0.995), higher Charlson Comorbidity Index (0.733, 0.673–0.798), having a higher baseline LDL > 160 mg/dL (0.753, 0.628–0.902), and having a male prescriber (0.872, 0.802–0.949). IMN was also less likely in patients with any ED visit (0.853, 0.764–0.952), hospitalization (0.787, 0.668–0.927), clinic visit (0.674, 0.513–0.885), or prescription (0.616, 0.546–0.695) in the past year than in those without a prior history of each healthcare utilization measure. While this study assessed whether statin use was for primary or secondary prevention of cardiovascular disease and found statistically more patients with primary prevention with IMN, this clinical characteristic was not reported in the multivariable logistic regression. No other studies reporting prevalence or associated factors provided information on whether the lipid lowering medication was being used for primary or secondary prevention.

In the second study, men were more likely than women to obtain their lipid-lowering medication [17]. Patients who were 55–64 years were less likely to obtain the medication than those 44 years and younger. Compared to patients with at least 9 prescription claims in the past 6 months, those in the 1–2 or none categories were also significantly less likely to obtain their lipid-lowering medication.

In the last study, the authors also used a multivariable logistic regression to compare patients with IMN to those with ongoing adherence [18, 19]. Patients with IMN (no prescription fills) were compared to those with at least 2 medications fills within 180 days. There was a higher likelihood of IMN in patients with Hispanic ethnicity (reference non-Hispanic White), less than 10 years of health-plan enrollment (reference 10 or more years), and comorbidity categories of three, and 4 (reference no comorbidity). If the lipid-lowering medication was not prescribed by primary care, there was a lower likelihood of IMN.

### Reasons for IMN (*n* = 4 studies)

Of the four studies reporting reasons for IMN to lipid-lowering medications, three were based on surveys [23–25] and 1 used focus group interviews (Table 2) [26].

**Table 2:** Studies reporting reasons for IMN to lipid-modifying medications (*n* = 4)

Study	Study design	Population	Location	Medication	Definition of IMN	Reasons for IMN
Harrison et al.	Semi-structured telephone interview	Sample of patients with IMN from a randomized, controlled trial (RCT) of an intervention to increase adherence to new statin Rx	Southern California	Statin	Not obtaining new statin medication within 12 weeks	Top reasons (*n* = 98) • 63% - Concerns about statin • 63% - Trying lifestyle modifications • 53.4% - Fear of side effects • 38.9% - Did not think needed statin • 34.7% - Disbelief condition was life-threatening
McHorney, et al.	Survey	Subset of patients with IMN from a national internet-based panel of adults with chronic disease, including dyslipidemia	National internet-based panel of adults	Lipid-lowering agents	Self-reported not filling a new medication for dyslipidemia within the past year	Top reasons (*n* = 79) • 64.6% - Fear of side effects • 44.3% - Financial hardship to pay for medication • 43% - Concerns about taking the medication • 26.6% – Did not think needed the medication
Tarn DM, Pletcher MJ, Tosqui R, et al.	Self-administered survey	Patients with IMN to statin Rx recruited from two academic health systems and through nationwide internet advertisements	California (academic health systems); Internet ads in Los Angeles and San Francisco, CA; Chicago, IL; Baltimore, MD; Detroit, MI; Jackson, MS; New York NY; and El Paso, TX	Statin	Self-report that had been prescribed a statin but did not take it	Reason for not filling (*n* = 99) or starting a statin (*n* = 74) • 27.2% - Worry about side effects • 26.6% - Want to try diet/exercise first • 16.8% - Prefer natural remedies/supplements • 15% - Want more testing
Tarn DM, Barrientos M, Pletcher, et al.	Focus group	Patients with IMN to statin Rx recruited from (1) academic medical center, (2) internet advertisements and (3) a large internet based cardiovascular cohort	California (academic medical center); Internet ads in 22 United States metropolitan areas; national cohort	Statin	Self-report that had been prescribed a statin in the past 2 years but did not take it	Major themes (*n* = 61): • Desire for alternative treatments • Worry about risks of statins • Perceptions of good personal health • Uncertainty about statin benefits

*Abbreviations* Rx, prescription

In one study, 98 participants receiving care in a managed care setting were randomly selected for a telephone survey inquiring about reasons for not filling their statin prescription [23]. About half the participants were men and 81% had at least a high school degree. 49% of participants were White, and 21.4% were Hispanic. At 12 weeks after the index prescription date, 74.5% of the participants had not obtained their new statin medication, 20.4% had picked it up from a setting other than the managed care setting, 4.1% noted they had already picked up the medication, and 1.0% were unsure whether the prescription had been filled. The top reported barriers for IMN included concerns about the statin (63%), and preference for lifestyle modifications (63%). Health literacy was a barrier in 32.9% of participants, with 29.6% lacking confidence when filling out health care forms, 17.1% having issues understanding their medical condition, and 16.9% noting they needed help reading medical information.

The second study was a survey of participants sampled from a national internet database of adults with chronic disease with IMN [24]. The survey included 79 participants with hyperlipidemia: their top reported reasons for IMN included fear of side effects (64.6%), followed by financial hardship related to paying for the medication (44.3%), and concerns with taking the medication (43.0%).

Another two studies were authored by the same group and employed a survey and focus group interviews to assess reasons for IMN [25, 26]. Participants with IMN to statin medications were recruited from two academic health systems and internet advertisements. A total of 173 respondents answered questions in a self-administered survey about their reasons and views about IMN for statin use [25]. The average age of the participants was 48.2 years, with the majority of the participants being White (62.8%). Forty-nine of 173 (28.3%) participants had a history of cardiovascular disease (CVD). Overall, 42.8% of participants picked up their medication but never took it, and the remaining 57.2% never obtained the medication. The main reasons for IMN to the statin medication were concern about the side effects (27.2%) and wanting to try exercise and diet first before taking a medication (26.6%). Those with a history of CVD reported concerns about side effects as their main reason (51%) compared with those without a history of CVD, who preferred exercise and diet first (33.9%) (*P* < .001). When asked specifically about their views on the risks of statins, 80.9% of participants noted they strongly or somewhat strongly had concerns about side effects. Additionally, 75.1% stated that they did not want to have to take a medication daily.

In the focus group interviews by the same author group, 61 individuals with IMN to statins participated [26]. Participants were recruited in a manner similar to the survey study. The themes from this study were similar to patient-reported reasons from the survey and included preference to try alternative lifestyle activities prior to medication, concern about side effects, and lack of perceived need for the medication.

### Outcomes (*n* = 1 study)

One study reported worse outcomes for patients from a managed care organization with IMN to newly prescribed lipid-lowering therapy (Table 3) [27]. IMN was defined as not having obtained the medication within 180 days of the prescription date. The authors found that the adjusted change in low-density lipoprotein (LDL) values from baseline to post-prescription was 41 mg/dL higher in patients with IMN compared to those who were adherent (*P* < .05). Adherence was assigned when the proportion of days covered (PDC) metric was at least 80%. Additionally, the hazard ratio of emergency department visits and hospitalizations (all-cause) was 1.25 (95% confidence interval, 1.04–1.50) for patients with IMN compared to patients deemed adherent.

**Table 3:** Studies reporting outcomes from IMN to lipid-modifying medications (*n* = 1)

Study	Study design	Population	Location	Medication	New Rx definition	Definition of IMN	Comparison	Results
Lee et al.	Retrospective cohort	Patients from a managed care organization with newly prescribed lipid-lowering therapy	California	Lipid-lowering agents	No prior fills for any cholesterol drug during the 12 months before the date the prescription was ordered	Not having obtained the medication within 180 days of the prescription date	Patients with IMN vs. patients deemed adherent (proportion of days covered ≥ 80%)	Low-density lipoprotein (LDL) • Adjusted change in LDL from baseline to post-prescription, 41 mg/dL higher in patients with IMN vs. adherent (*P* < .05). Emergency department visits and hospitalizations (all-cause) • Hazard ratio of 1.25 (95% confidence interval, 1.04–1.50) for patients with IMN vs. adherent

### Interventions to address IMN (*n* = 4 studies)

Four studies assessed interventions to prevent or address IMN using automated and/or live telephone calls by a pharmacist or nurse (Table 4**)** [28–31]. Only one of these studies focused solely on lipid-lowering medications [28].

**Table 4:** Studies reporting interventions for IMN to lipid-modifying medications (*n* = 4)

Study	Study design	Population	Location	Medication	New Rx definition	Intervention	Control	Measurement of IMN	Results
Derose et al.	RCT	Patients from an integrated healthcare system who did not fill a new statin after 1 to 2 weeks	California	Statin	No Rx claim for a statin within the past year	Automated telephone calls followed 1 week later by a letter for continued nonadherence (*n* = 2,606)	Usual care (*n* = 2,610)	No statin dispensed up to 2 weeks after the expected delivery of the letter (32 − 29 days after Rx).	57.7% (1,504/2,606) intervention vs. 74% (1,931/2,610) control (*P* < .001)
Fischer et al., 2014	Retrospective cohort	Patients who had not picked up their new antihyperlipidemic from a community pharmacy chain		Lipid-lowering	No Rx claims for a medication in same therapeutic class within prior 6 months	Two sequential interventions: Automated phone calls on the 3rd and 7th days after Rx processing (*n* = 328,323 Rxs) Live phone call from a pharmacist or technician on 8th day after Rx processing (n = 51,254 Rxs)	Patients born on randomly selected birthdays received usual care (*n* = 3,504 Rxs for comparison to automated phone calls and *n* = 1,284 Rxs for comparison to live call)	No Rx claims for the medication or another in the same therapeutic class within 30 days of Rx processing at the pharmacy	Automated calls: 6% (19,544/328,323) intervention vs. 6% (210/3,504) control (*P* = .93) Live call: 39.4% (20,180/51,254) intervention vs. 40.8% (524/1,284) control (*P* = .25)
Fischer et al., 2015	RCT	Patients from primary care practices of an integrated healthcare delivery network who did not pick up a newly prescribed medication for hyperlipidemia within 14 days	Pennsylvania	Lipid-lowering	No Rx of the medication or another in same subclass within the prior year	Phone call from a nurse working with the prescriber (*n* = 19)	Usual care (*n* = 14)	Patients who did not pick up their hyperlipidemia medication from the pharmacy within 30 days	5.3% (1/19) intervention vs. 33.3% (5/14) control (*P* = .03)
O’Connor et al.	RCT	Patients with diabetes prescribed a new medication for uncontrolled LDL from multispecialty medical groups	California, Pennsylvania, Wisconsin or Washington	Lipid-lowering	No fill of medication class within prior 180 days	One scripted phone call from a nurse health manager, diabetes educator /trainee or pharmacist (*n* = 348)	Usual care (*n* = 315)	Medication not picked up within 60 days of prescribing	20.4% (61/299) intervention vs. 18.1% (49/270) control (*P* = .47

*Abbreviations* BP, blood pressure; EHR, electronic health records; e-Rx, electronic prescription; LDL, low-density lipoprotein; RCT, randomized controlled trial; Rx, prescription

Two randomized controlled trials (RCTs) found a significant benefit of the intervention on IMN. In the Derose et al. study, patients who had not picked up their newly prescribed a statin after 1–2 weeks were randomized to an automated phone call followed by a letter a week later [28]. Participant demographics were similar among the intervention and usual care groups in term of age, sex, race/ethnicity, spoken language, household income, and educational level. The mean age was around 56 years; about half of the participants were women; 30% were White, and 10% were Black. There were 30% Hispanic. The intervention group included an additional 16.3% of patients who picked up their prescription (*P* < .001). Factors associated with a higher likelihood to have a statin dispensed regardless of intervention or usual care group were Spanish vs. English speaking (OR, 1.32; 95% CI, 1.06–1.65; *P* = .01) and having a pharmacy drug benefit vs. none (OR, 10.05; 95% CI, 6.85–14.75; *P* < .001).

In the other positive RCT study, the intervention, a call from a nurse at the provider’s office, was in addition to standard procedure for the pharmacy chain of automated calls on the third and seventh days and a live call between days 10 and 14 for medications not picked up [30]. The effect of the standard pharmacy procedure was assessed in a retrospective cohort study and no effect compared to usual care was found [29]. The nurse call resulted in a difference in IMN of 28% favoring the intervention group over usual care (*P* < .03).

The RCT by O’Connor et al. failed to find a benefit of a call from a diabetes educator or clinical pharmacist from the medical group over usual care after prescribing of a medication for uncontrolled LDL [31]. Baseline characteristics of age, sex, race, drug coverage and mean LDL values were similar among the groups. For the adherence analysis, only individuals with drug coverage and at least 60 days were included, decreasing the participant number in the intervention group to 299 (from 348) and the control group to 270 (from 315). Before the intervention, several patients had already obtained their medication: 60.9% and 70% in the intervention and control groups, respectively. After the intervention, prescription fill rates increased to 79.6% in the call group and 81.9% in the usual care group (*P* = .47). No significant difference in LDL was found between the groups (mean change from baseline: -30.4 mg/dL intervention [*n* = 288] vs. -33 mg/dL control [*n* = 251], *P* = .44), although the study was underpowered to detect a difference in this measure.

## Discussion

This research summarizes the evidence on the prevalence, predictors, outcomes, and solutions of IMN for newly prescribed lipid-lowering medication. Prevalence was the most studied parameter, with estimates varying due to differences in study populations, data sources, and the definitions of a new prescription and IMN. Differentiating a new prescription from a new medication is important since patients may receive a new prescription for a continuing medication. A lower IMN was found in one study when the new prescription definition included both new and continuing medications [15]. All but one [20] of the prevalence studies in our systematic review helped to ensure that the prescription was a new medication by excluding patients with prior claims for the medication or class for a specified timeframe of 6 months to 1 year before the prescription.

The prevalence estimates also depend on the definition of IMN, which was defined as not obtaining the first fill of either the medication or one in the same class after a new prescription. Pharmacy records and claims data were used to determine this outcome. However, these data sources would not identify patients who obtained the initial fill of the medication but never took a dose. Studies could report the percentage of patients who obtained only one fill of the medication, but that measure may also include patients who took the medication and experienced treatment failure or adverse effects and did not obtain a refill. One study found that 3% of patients obtained only one fill of the medication in the first 180 days [18, 19]. In another study which only included participants with IMN to statin medications, 42.8% of them picked up their medication but never took it [25]. The intent of identifying IMN should include individuals who never take a dose of a newly prescribed medication but most studies have not captured individuals who obtain the medication but do not take any [32]. Including these individuals would increase the prevalence of IMN. Pharmacy records and claims data allow for identification of those with only one fill of the medication, but additional data sources would be needed to assess whether patients took any of the medication.

Based on the prevalence estimates, clinicians can expect to encounter IMN at least once in every 6 to 10 patients when they prescribe a new lipid-lowering medication.

Factors associated with IMN are related to the prescriber and the patient. Hispanic ethnicity and Black race, compared to non-Hispanic white rate were associated with IMN. These differences contribute to health inequity and must be addressed. Since no studies were designed to specifically examine race and ethnicity in relation to IMN, we recommend future research in this area.

Prescriptions by primary care providers were also associated with higher likelihood of IMN. Alongside this, IMN is more likely in patients with lower Charlson Comorbidity Index and lower use of healthcare system (i.e., no ED visits, hospitalization). These findings suggest that patients with less severe disease such as those using lipid lowering for primary prevention may be at higher risk for IMN. This aligns with some of the reasons patients mentioned for IMN such as a lack of perceived need for the lipid lowering medication and a disbelief about the threat of their condition. Patients having had a prior cardiovascular event and seeing a cardiologist may perceive a greater value of the medication.

To improve the gap in care that occurs for IMN, clinicians can implement strategies to prevent further delays in care. Certainly, from a research perspective, the pervasiveness of electronic prescribing has facilitated the identification of IMN. E-prescriptions are linked to pharmacy claims data identifying newly prescribed medications. However, this technology also allows clinicians to receive notifications when the medication is not obtained. The currently mandated electronic prescribing standard NCPDP SCRIPT Standard Version 2017071 contains this capability [33]. When a medication is prescribed, the clinician can request a fill status notification, termed “RxFill.” The pharmacy electronically notifies the clinician with one of several responses: the medication was picked up, the medication was not picked up, the medication was partially filled, or the medication was transferred to another pharmacy.

The challenge is that there have been delays in making this application in e-prescribing software easily accessible and integrated into health care workflows. Currently, the ability to request a fill status notification (i.e., check the RxFill box), is not available or readily seen on the e-prescribing screens. Clinicians and administrators can ask their e-prescribing vendor to turn on and integrate this feature into their typical prescribing workflow.

Additional support for using this technology comes from the Centers for Medicare and Medicaid Services (CMS). As of January 2022, using the electronic prescribing standard NCPDP SCRIPT Standard Version 2,017,071) is required when e-prescribing for beneficiaries in the Medicare prescription drug benefit (Part D) program [34]. CMS has signaled further support for technology advanced with the proposed rule which is anticipated to require a newer version of the NCPDP SCRIPT standard (version 2,023,011) [35].

These measures can improve clinician awareness of IMN, as there is concern that individuals who do not fulfill the initial prescription may be less likely to return to the prescriber for follow-up or inform providers of their decision. In the focus group interviews that identified patient-reported themes for IMN, one-third of 61 participants noted that they did not tell their provider of their decision [26].

One of the main reasons that patients explained for their IMN was concern about the side effects from the statin medication and wanting to try lifestyle medications. This is similar to what is found in a registry study [36]. In patients eligible for statin therapy and offered a statin, 5% of primary prevention patients and 2% of secondary prevention patients reporting declining the treatment. The most common reason given at 36.8% (overall for both primary and secondary prevention groups) for declining treatment was worry about side effects. The authors discuss that these individuals appear more concerned with side effects of statins rather than CVD. Discussing potential harms of statin therapy may be helpful and guidance on providing simple language when discussing risk includes talking about absolute risk and not providing estimates of a single person as in “1” out of 10 [37]. Additionally, asking patients whether they are willing to begin a lipid-lowering medication may elicit the need for a discussion about side effects or other aspects such as wanting to try lifestyle first.

Patients with IMN to lipid-lowering therapy delay their reduction in cardiovascular risk and there is a suggestion of worse clinical outcomes [27]. The economic impact and patient-reported outcomes of IMN are less well understood. Understanding the trajectory of IMN on the future use of lipid-lowering medications and overall patient care will be important to improve health care delivery models.

### Clinical implications summary

IMN to lipid-lowering therapy may be more common than perceived in clinical practice, especially when patients with IMN do not return for follow-up care. Before prescribing a new lipid-lowering medication, ask the patient what their concerns are as the primary reasons given for IMN were side effects and wanting to try lifestyle modifications first.

Afterwards, ensure follow-up care as prescribers generally remain unaware that their patient has not obtained the medication until the patient returns for a subsequent office visit.

### Limitations

The result of any systematic review is highly dependent on the strength of the search protocol and the reviewers’ decisions for determining whether studies meet the inclusion and exclusion criteria. The medical literature was searched using PubMed and Google Scholar but did not include other databases. EMBASE was not searched because the inclusion criteria specified IMN within US populations, and resources did not allow for review and analysis of the many non-US populations included in this database. A post hoc search of the Cochrane Collaboration did not reveal any publications addressing IMN, only those on ongoing medication adherence. This is not surprising considering the small number of IMN studies included in this review, and only four studies addressing interventions to improve IMN.

During screening, articles on secondary nonadherence were excluded which may have missed studies that included IMN as a secondary outcome.

The other inherent limitation is that the prevalence of IMN to lipid-lowering agents depends on the populations studied and the definition of IMN; therefore, the ranges reported must be interpreted with caution. Additionally, due to the varied definitions of IMN and timeframes in which IMN was assessed in the included studies, no synthesis of IMN prevalence was conducted.

Given the paucity of literature on this topic, the purpose of this review was to provide a comprehensive summary of all studies in the United States that addressed IMN to lipid-lowering agents. A risk of bias assessment was not performed. As such, studies of low quality may have been included. For the intervention studies, there were concerns with small sample sizes [30] and the inclusion of patients who picked up their medications before the intervention [31].

## Conclusions

Initial medication nonadherence to lipid-lowering therapy is common, with up to 20% of patients not obtaining their prescribed medication. Patients report being concerned about the side effects of statins and wanting to first try lifestyle interventions. Interventions to address IMN show mixed results. Future research should determine efficient and effective approaches to prevent and address IMN.

## Data Availability

All data generated or analyzed during this study are included in this published article.

## Abbreviations

CI: Confidence interval
CMS: Centers for Medicare and Medicaid Services
IMN: Initial medication nonadherence
LDL: Low-density lipoprotein
OR: Odds ratio
